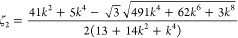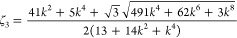# Pattern Selection in Three-Precipitate Liesegang Systems

**DOI:** 10.1021/acsomega.4c05735

**Published:** 2024-10-18

**Authors:** Tamar Kanimian, Pamela Nasr, Rabih Sultan

**Affiliations:** † Department of Chemistry, American University of Beirut, PO Box 11-0236 Riad El Solh, Beirut 1107 2020, Lebanon; ‡ Department of Chemistry and Chemical Biology, Cornell University, Ithaca, New York 14853-0001, United States

## Abstract

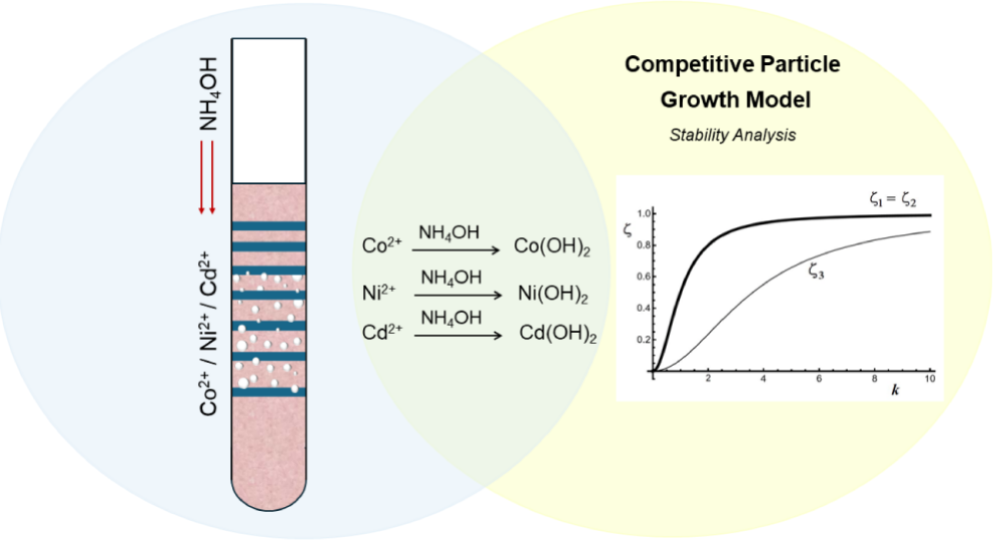

Liesegang patterns present a display of parallel stripes of precipitate that arise from the interdiffusion of coprecipitate ions in a gel medium. The bands observed in rocks are typical analogies of this phenomenon, and their composition is not restricted to the banded deposition of a single mineral. We here extend the study to a three-precipitate system, wherein Co^2+^, Ni^2+^, and Cd^2+^ cations are precipitated by the same anion (OH^–^ from NH_4_OH) to form Co(OH)_2_, Ni(OH)_2_, and Cd(OH)_2_, respectively. The resulting pattern exhibits an alternation of compact mixed Co(OH)_2_ and Ni(OH)_2_ bands with granules of pure Cd(OH)_2_ between them. The obtained pattern confirms the generic type of (*A* + *B*)/*C*/(*A* + *B*)/*C*/(*A* + *B*)/*C* alternation conjectured analytically using the competitive particle growth (CPG) model and stability analysis.

## 1 Introduction

Liesegang bands or rings are visual structures in space that arise from the coupling between diffusion and a precipitation reaction in a gel medium.^1−4^ Besides being the product of a multitude of laboratory experiments, Liesegang patterns find their most familiar similarity in the banded scenery observed in a vast variety of rocks.^5−7^ Research on this rich dynamical phenomenon encompassed a wide range of modifications, such as, to name but a few, exploring a wide choice of precipitating salts,^8,9^ using different gel materials,^11−13^ conducting gas phase reactions,^14^ coupling to complex formation,^15,16^ imposing applied physical vectors,^17,18^ and most notably and extensively, theoretical modeling.^19−21^ One additional variant of interest and relevance in this study, is the simultaneous presence of more than one precipitating salt.^22,23^

When Co(OH)_2_ is precipitated from Co^2+^ with concentrated NH_4_OH, the precipitate can redissolve due to complex formation with NH_3_. In a Liesegang framework, this is manifested in a scenario of band formation at the bottom of the pattern and band dissolution at the top. The two reactions are
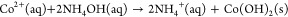
1
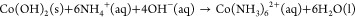
2

The dynamic evolution of the Co(OH)_2_ system could be altered by the introduction of a second cation. In a previous study,^24^ a suitable cation was chosen in such a way that the complex formation with ammonia, and hence the dissolution of its hydroxide, would dominate over the dissolution of cobalt hydroxide at the interface. Ni^2+^ is an appropriate cation since (1) the solubility product constant (*K*_sp_) of Ni(OH)_2_ is similar to that of Co(OH)_2_; and, (2) the formation constant (*K*_f_) for the Ni(NH_3_)_6_^2+^ complex is 4 orders of magnitude higher than that of the Co(NH_3_)_6_^2+^ complex. The values are reported in Table 1.

**Table 1:** Solubility Product Constants of the Hydroxides and Formation Constants for the Corresponding Ammonia Complexes^25^

Salt	*K*_sp_ of the salt	*K*_f_ for the ammonia complex
Co(OH)_2_	1.6 × 10^–15^	5.0 × 10^4^
Ni(OH)_2_	2.0 × 10^–15^	2.0 × 10^8^
Cd(OH)_2_	2.5 × 10^–14^	-

Whereas the nickel-free system exhibited regular Liesegang bands of Co(OH)_2_, a cutoff concentration was detected in the presence of nickel ions, above which only a uniform continuous precipitation zone propagating down the tube was obtained. Below that threshold, a regular periodic pattern was observed. The velocity of the advancing front decreased with an increase in the concentration of the competing ion. As the concentration of Ni^2+^ increases, the dissolution at the upper region is inhibited and the precipitation at the lower portion is enhanced. A number of two-precipitate systems were studied in ref.^23^ The main interest lies in the dynamical morphology of the pattern, i.e., whether we obtain an overlap (correlated pattern) or an alternation (anticorrelated pattern). A theoretical criterion was developed,^22,23^ based on a stability analysis of the dynamics, to delineate the distinction between the two types. In the same study,^23^ the PbI_2_–PbF_2_, Ag_2_Cr_2_O_7_–PbCr_2_O_7_, MnS-CdS, and MnS-CuS systems were tested experimentally, and classified accordingly.

In this paper, we extend our curiosity to three-precipitate systems. It is to be noted that different sets of precipitates yield different behaviors, and no generic trend is observed. Focusing on the Co(OH)_2_–Ni(OH)_2_ system, the mere introduction of Mg^2+^ as a third inner electrolyte drastically modified the morphology in an unpredictable way. A highly rhythmic pattern, characterized by the formation of band multiplets with ascending number of bands within them was obtained.^15^ At all concentrations of Mg^2+^, pattern formation was initiated by the appearance of white magnesium hydroxide bands near the interface. Beyond this succession of Mg(OH)_2_ bands, the pattern of ascending rhythmicity appeared, with bands consisting solely of Co(OH)_2_ and Ni(OH)_2_ with no magnesium hydroxide. Outside a range of [Mg^2+^] (0.20 M–0.75 M), the strange rhythmicity ceases, and a classical periodic pattern is observed.

In the present study, we modify the identity of the “third” foreign cation, i.e., we keep Co^2+^ and Ni^2+^, but we select Cd^2+^ in lieu of Mg^2+^. A whole new set of dynamical properties is obtained, revealing the diversity, complexity, and wealth of this physicochemical system. We first present a theoretical model based on a stability analysis approach of the “triad” system, within the competitive particle growth (CPG) model,^20,26^ and compare theory with experiment.

## 2 Stability Analysis

Our system involves metal hydroxide precipitates Co(OH)_2_ (*A*), Ni(OH)_2_ (*B*), and Cd(OH)_2_ (*C*). It can be represented by three chemical equations coupled through the hydroxide ion OH^–^ (denoted here by *Z*), as follows:
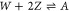
3
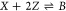
4
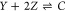
5where
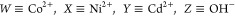


Several models were considered to simulate complex Liesegang patterns. They are grouped into two major categories (with further subdivisions): prenucleation theories^19,27^ and postnucleation theories.^26,28^ The former category is based on the Ostwald supersaturation–nucleation–depletion cycle,^29^ while the latter focuses on a symmetry breaking instability of a uniform colloidal sol mediated by surface tension and the coarsening of precipitate particles (the so-called Ostwald ripening phenomenon).^30,31^ This latter mechanism leads to the spatial distribution of precipitate and precipitate free domains, or bands in 1D. Some models combine the two concepts, incorporating both nucleation and particle growth into the dynamical equations.^21,32,33^ When two or more precipitates are involved, we can show^22,23^ that nucleation is not a necessary requirement for the pattern selection (overlap or alternation). The latter is essentially determined by the competitive particle growth between the various precipitates.

Interactions between the ions and the gel are, also, undoubtedly important as was emphasized by a number of studies.^34,35^ Such interactions were modeled theoretically, notably for Ca^2+^ with agarose,^36^ and Ag^+^ and H^+^ with gelatin.^37^ It was found^36^ that Ca^2+^ renders the gel coarser notably with [Ca^2+^] < 0.20 M. A general trend from hydrodynamic and molecular models is thus the significant decrease in the diffusion coefficient of the ion-solute, sometimes more than 2 orders of magnitude.^38^ Such a large reduction suggests that electrostatic forces could bring about the immobilization of charged particles.^38^ This overall decrease does not affect the nature of the dynamics of diffusion–precipitation coupling and only governs the choice of the diffusivity value. Horkay^39^ established that in fully neutralized polyelectrolyte gels, in the presence of added salt, the ionic term is not expected to play an explicit role, although ionic interactions may modify the mixing free energy contribution. This realization applies in our gelatin–Co^2+^–Ni^2+^–Cd^2+^ system, wherein the measured pH was 6.79 (experiment reported in Figure 3). Electrolyte–polymer gel interactions play a central role in the modeling when a pattern other than Liesegang bands is formed. The formation of dendritic patterns instead of a Liesegang pattern in PbF_2_ systems when the role of the electrolytes was reversed (F^–^ up and Pb^2+^ down in the gel, in lieu of the reverse), was attributed^40^ to random diffusion due to electrostatic repulsion between F^–^ and the negative groups in the agar gel.

We now write the evolution equations for the three precipitates and the ionic species. Let *R*_*A*_, *R*_*B*_, and *R*_*C*_ be the particle radii of precipitates *A*, *B*, and *C* respectively. The particle growth kinetics is, therefore
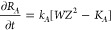
6
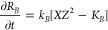
7
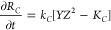
8where *k*_*A*_, *k*_*B*_, and *k*_*C*_ are precipitation rate constants, and *K_A_*, K_B_, and *K_C_* are dissolution equilibrium constants. The evolution equations (diffusion-precipitation) for the aqueous species *W*, *X*, *Y,* and *Z* are
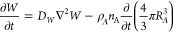
9
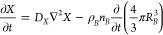
10
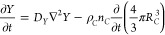
11
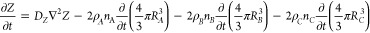
12where ρ and *n* are the molar density and the number density respectively, and *D* is the diffusion coefficient of the aqueous species. The particles are assumed to be spherical, which explains the term , representing the volume of the particle.

We introduce linear perturbations for each the seven variable of the form  (and same for *R*_*B*_ and *R*_*c*_),  (and same for *X*, *Y* and *Z*), where  and  are the steady-state values. Changing to dimensionless perturbations for the radii , ψ_B_ = δR_B_/ (bar) and , and the aqueous species , , , and , and the time τ, the evolution equation for the scaled perturbation ψ_*A*_ becomes
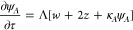
13where , and κ_*A*_ is a key parameter in the competitive particle growth (CPG) theory, which is a measure of the dependence of the dissolution constant *K* on particle size, defined by
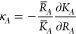
14and similarly for ψ_*B*_ and ψ_*C*_. The details of the method are presented in ref.^23^ The scaled perturbations ψ_*A*_, ψ_*B*_, and ψ_*C*_, and *w*, *x*, *y*, and *z* are analyzed in terms of Fourier modes of the form:
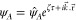
where  is the perturbation amplitude, ζ the stability eigenvalue and  the wave vector.

By substituting in the evolution eqs 6–12, and setting all scaled parameters equal to 1 except*κ*_*A*_, κ_*B*_, and κ_*C*_, we obtain the following set of equations:
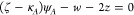
15
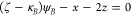
16
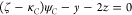
17
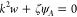
18
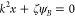
19
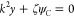
20
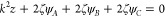
21

Taking κ_*A*_ = κ_*B*_ = κ_*C*_ = 1 as a first set and setting the determinant of the coeffcients matrix for the seven variables equal to zero, we obtain the following three solutions for the stability eigenvalue ζ:
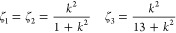
22

**Figure 1:**
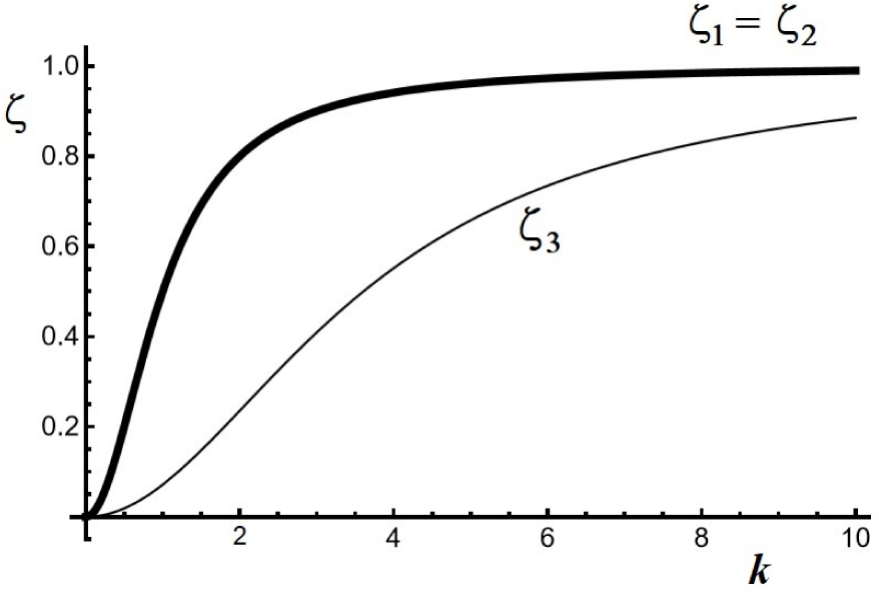
Plot of stability eigenvalue ζ versus wave vector *k*, for a first set of κ values: κ_*A*_*=* κ_*B*_*=* κ_*C*_*=* 1. The fastest growing mode (ζ_1_*= ζ*_2_) corresponds to a situation where the perturbations are related by ψ_*C*_*= –* (ψ_*A*_ + ψ_*B*_), indicating the formation of an anticorrelated pattern of the form (*A* + *B*)/*C*/(*A* + *B*)/*C*.

Figure 1 shows a plot of ζ versus *k*, showing that ζ_1_ (=ζ_2_) indicates a faster growing mode than ζ_3_.

**Figure 2:**
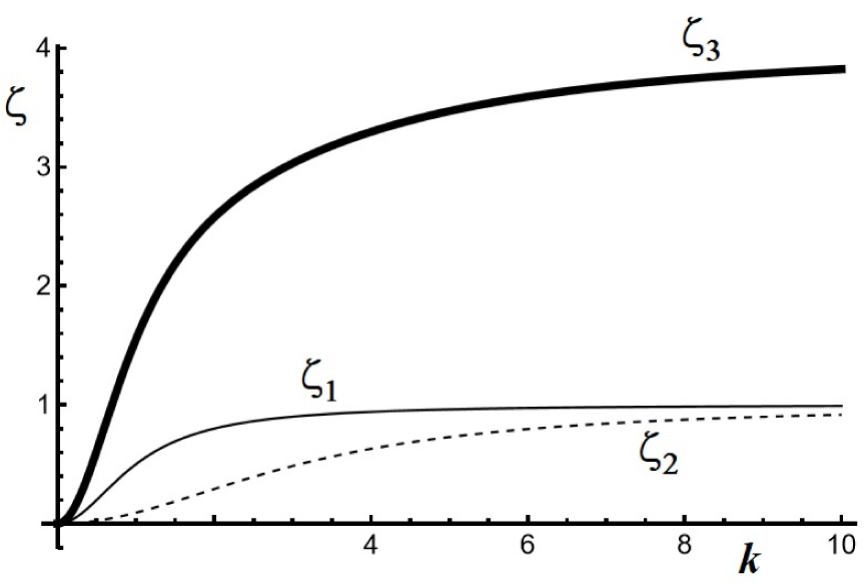
Plot of stability eigenvalue ζ versus wave vector *k*, for a second set of κ values: κ_*A*_*=* κ_*B*_*=* 1; κ_*C*_*=* 4. The fastest growing mode (ζ_3_) corresponds to a situation where the perturbations are related by ψ_*C*_ = −(ψ_*A*_ + ψ_*B*_), indicating the formation of an anticorrelated pattern of the form (*A* + *B*)/*C*/(*A* + *B*)/*C*.

This result is of fundamental importance because ζ_1_ corresponds to a growth mode where perturbations ψ_*A*_, ψ_*B*_, and ψ_*C*_ are related by ψ_*C*_ = −(ψ_*A*_ + ψ_*B*_). Such a mode leads an anti-correlated pattern wherein we have bands containing overlapping precipitates *A* and *B*, alternating with regions of single precipitate *C* of the form: (*A* + *B*)/*C*/(*A* + *B*)/*C*/(*A* + *B*)/*C*··· The other mode represented by ζ_3_ corresponds to a situation where ψ_*A*_ = ψ_*B*_ = ψ_*C*_, leading to a pattern wherein the three precipitates overlap within the same band. We call this a correlated pattern. Since ζ_1_ = ζ_2_ > ζ_3_ for all *k*, the pattern selection reposes on the anticorrelated pattern. Figure 2 illustrates another calculation, where *κ*_*A*_ = κ_*B*_ = 1 and κ_*C*_ = 4.

In this case, three different solutions for ζ are obtained (expressions reported in the Appendix). Here, the fastest growing mode corresponding to ζ_3_ has *ψ*_*C*_ =̵(*ψ*_*A*_ + *ψ*_*B*_), and hence also leads to an alternaing pattern of the form (*A* + *B*)/*C*/(*A* + *B*)/*C*/(*A* + *B*)/*C*···.

## 3 Experimental Section

We now carry out Liesegang experiments in gelatin involving three precipitates: Co(OH)_2_, Ni(OH)_2_, and Cd(OH)_2_.

### 3.1 Procedure

A sample of gelatin powder (Difco) was weighed and added to deionized water to obtain 5% gelatin gel (w/w H_2_O). Samples of the inner electrolytes 0.20 M CoCl_2_.6H_2_O (Sigma-Aldrich), 0.10 M NiSO_4_.6H_2_O (Mallinckrodt), and selected concentrations (0.10 M, 0.20 M, 0.30 M, 0.40 M, and 0.50 M) of CdCl_2_·2.5H_2_O (Baker Analyzed Reagents) were added to the water gelatin mixture. The said mixture was then heated with stirring to 95 °C and then poured into thin glass tubes (of length 18 cm and inner diameter 5.0 mm) until two-thirds full. Then, the tubes were covered with Parafilm and allowed to gel for 24 h. After gelation, 11.50 M NH_4_OH (Fluka) was added to the remaining one-third of the tube. After the junction between the outer electrolyte and the solidified gel was marked, the tubes were placed in an air thermostat at 18 °C ± 1 °C. Images of the tubes were captured every 3–4 days over 16 days, using a digital camera (Sony Cyber Shot DSC-QX30 operated by a Samsung J1 cell phone through the Playmobile application) while mounted on a movable stand.

### 3.2 AAS Sample Preparation

**Figure 3:**
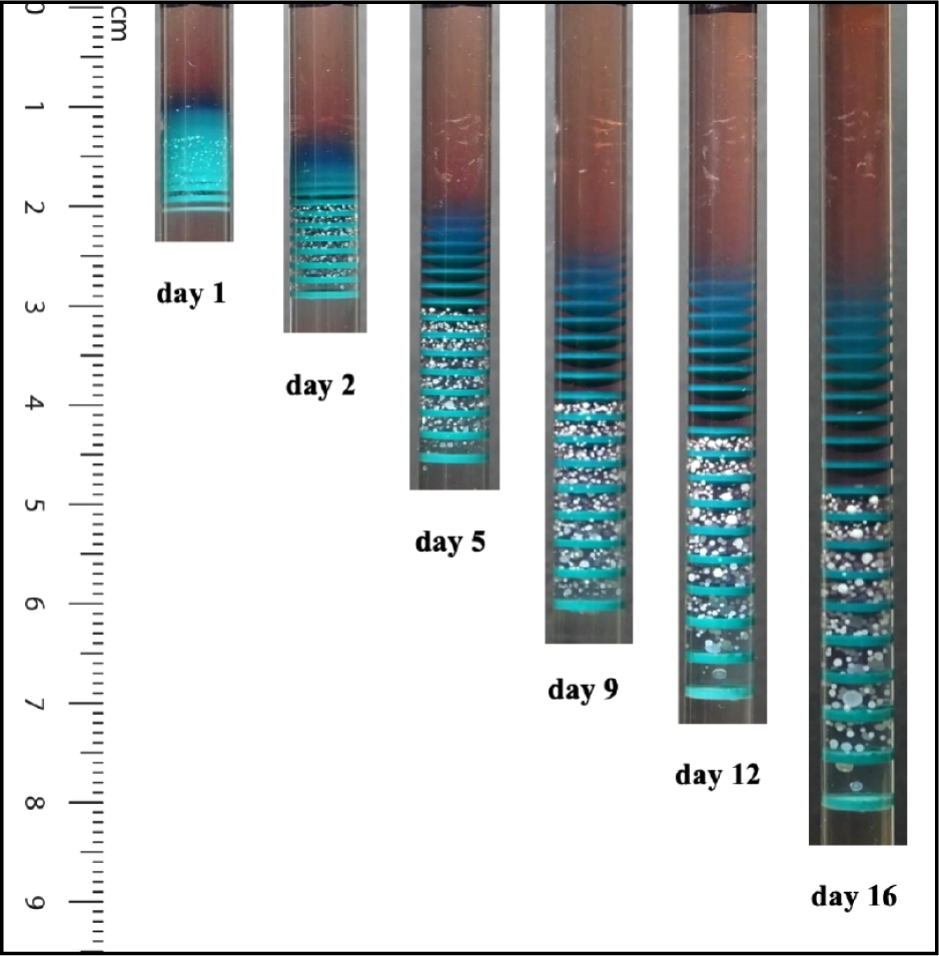
Pattern of bands/granular particles with time evolution. The time (in days) is indicated below each image. [Co^2+^]_0_ = 0.20 M; [Ni^2+^]_0_ = 0.10 M; [Cd^2+^]_0_ = 0.20 M; [NH_4_OH]_0_ = 11.50 M. The bands consist of mixed Co(OH)_2_ and Ni(OH)_2_, while the particles are uniquely Cd(OH)_2_. (Photograph courtesy of Tamar Kanimian, Copyright 2024).

The composition of the bands and interband regions in the metal elements Co, Ni, and Cd was measured using flame atomic absorption spectrophotometry (AAS) (SOLAAR Atomic Absorption). In AAS, the sample is broken down into individual atoms in the flame, at a very high temperature. The characteristic absorption wavelengths of the three elements Co, Ni, and Cd are 240.7, 232.0, and 228.8 nm, respectively. To prepare the samples for analysis, the tube is carefully cut from the bottom, and the gel is allowed to slide out on a glass plate. The band regions to be studied are then meticulously cut at the edges and placed in clean vials. To each sample was added 0.30 mL of 1.0 M HCl (VWR chemicals) with stirring until the precipitate band (hydroxide) is completely dissolved. After that, 2.0 mL of deionized water is added to dilute the solution. As further dilution was necessary to nest the concentration, 0.10 mL of the latter solution was again diluted to 25.0 mL. For Cd(OH)_2_, which appears as granules (Figure 3), the latter are carefully isolated from the gel, washed thoroughly and then treated with HCl, just like the bands. Due to the low solubilities of Co(OH)_2_, Ni(OH)_2_, and Cd(OH)_2_ (*K*_sp_s reported in Table 1), it is assumed that all the cations from the gel are precipitated as their hydroxides.

The relative absorbances were converted to weight percentages of the hydroxides against a calibration curve by using known standards.

### 3.3 Results

Figure 3 shows the obtained patterns with time monitoring for 16 days.

**Figure 4:**
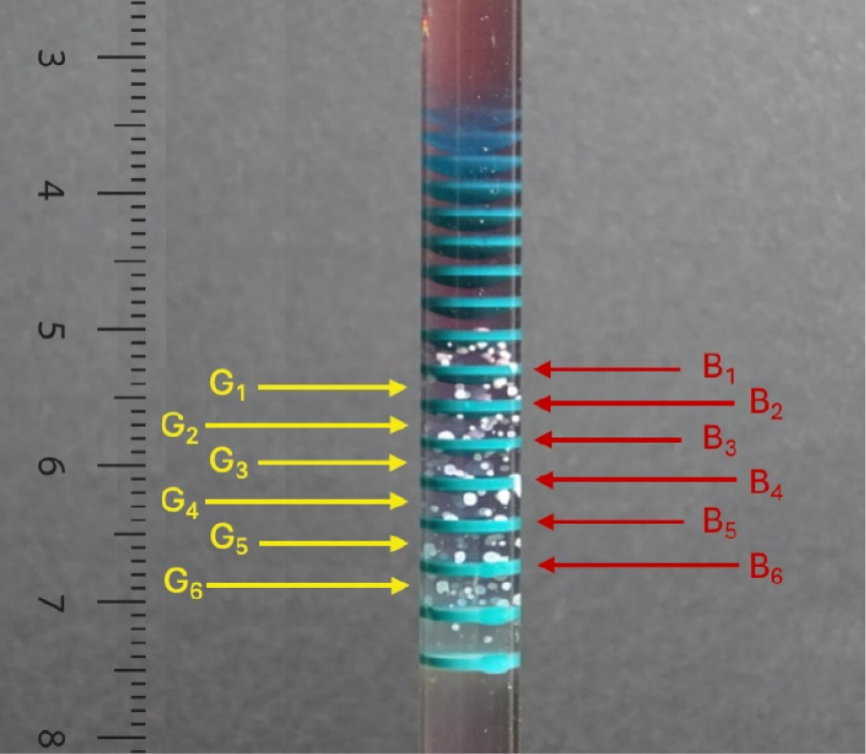
Tube dissected for analysis. Bands B_1_–B_6_ and granular particles G_1_–G_6_. Same experiment as that in Figure 3. Analysis performed at day 11. (Photograph courtesy of Tamar Kanimian. Copyright 2024).

We see a distinct alternation of compact bands and granular particles zones. As we move down the tube, the bands veer in color from dark blue to greenish light blue, which is a preliminary visual indication of gradual increase in Ni(OH)_2_ content. The darker blue color of the top bands points toward a higher percent composition in α-Co(OH)_2_. This feature and the gradual color alteration are confirmed by flame atomic absorption spectrophotometry (AAS). Figure 4 highlights the different zones (compact bands and interband gel with granular particles) to be analyzed by AAS. The percent compositions of the bands B_1_ through B_6_ are recorded in Table 2.

**Table 2:** Percent Composition in Co(OH)_2_ and Ni(OH)_2_ in Bands B_1_ through B_6_ of Figure 4

Band	Co(OH)_2_%	Ni(OH)_2_%
B_1_	78.1	21.9
B_2_	77.8	22.2
B_3_	76.2	23.8
B_4_	73.6	26.4
B_5_	71.6	28.4
B_6_	69.0	31.0

The measurements confirm the gradual decrease in percent Co(OH)_2_, and increase in percent Ni(OH)_2_. As discussed in the Introduction, the preferential dissolution of Ni(OH)_2_ results in higher Co(OH)_2_ precipitate content in the top bands, and then the latter decreases as we go to the lower bands where the Ni(OH)_2_ composition catches up by gradual increase. Analysis of the interband scattered particles in gel regions G_1_–G_6_ establishes that they consist almost exclusively of Cd(OH)_2_. Only infinitesimal traces of Co^2+^ and Ni^2+^ were revealed, believed to result from adsorption on the precipitate particles.^10^ Such ionic remnants could also originate from attractions by partially negative oxygens and nitrogens^41,42^ (carbonyl, carboxyl, amino, and imine groups) from the gelatin. The gel is washed off, and some Co^2+^ and Ni^2+^ remain adherent in traces. The presence of the latter ions could not emerge from the dynamical mechanism, as it would certainly not appear in traces but in appreciable amounts, comparable with Cd^2+^. This is in complete harmony with the differentiating model analysis of Section 2. Hence, an important result of this study is the pattern selection inherent in the dynamics. The alternation Co(OH)_2_–Ni(OH)_2_ bands/Cd(OH)_2_ particles exactly matches with the anticorrelated pattern, found to be dominant in the linear stability analysis of Section 2.

**Figure 5:**
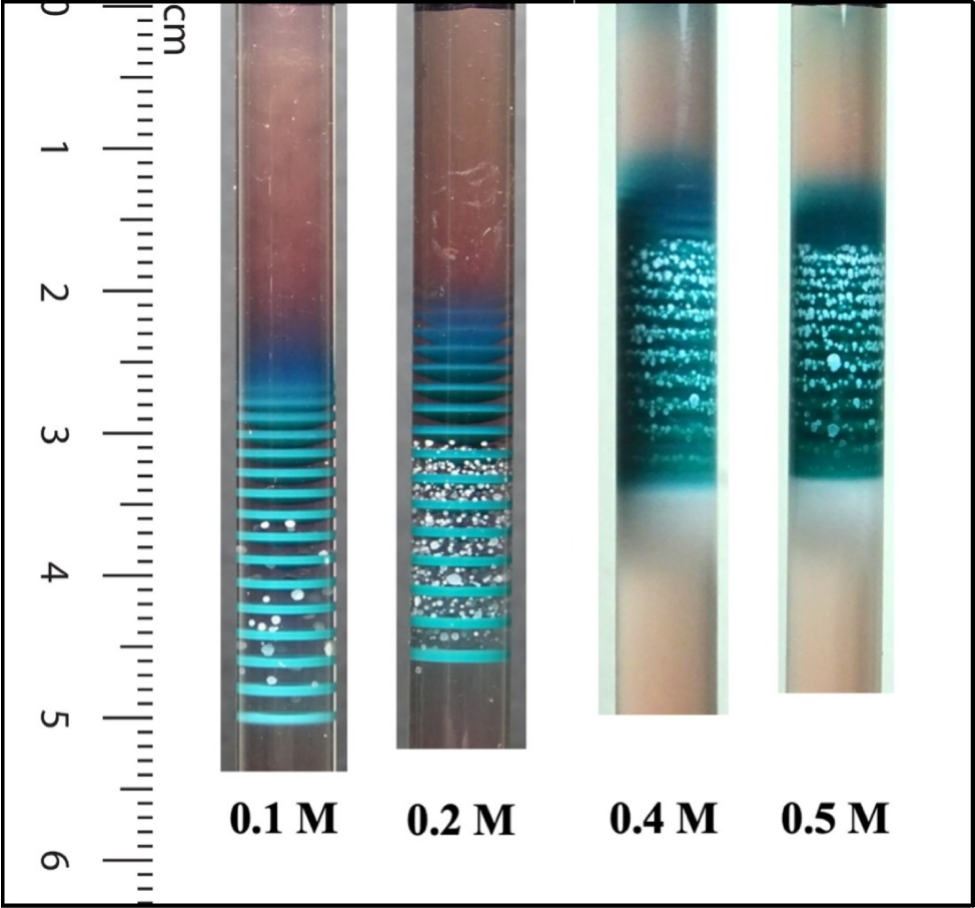
A set of experiments at different Cd^2+^ initial concentrations, varied as a control parameter, and indicated under each tube. (Photograph courtesy of Tamar Kanimian. Copyright 2024).

Figure 5 depicts the alternation between bands and granules at different concentrations of the Cd^2+^ ion as a control parameter.

We see that the number density of the Cd(OH)_2_ particles increases between the Co(OH)_2_/Ni(OH)_2_ compact bands, as [Cd^2+^]_0_ increases. The interesting observation here is that the pattern maintains the anticorrelated (alternating) characteristic.

## 4 Geological Analogies

**Figure 6:**
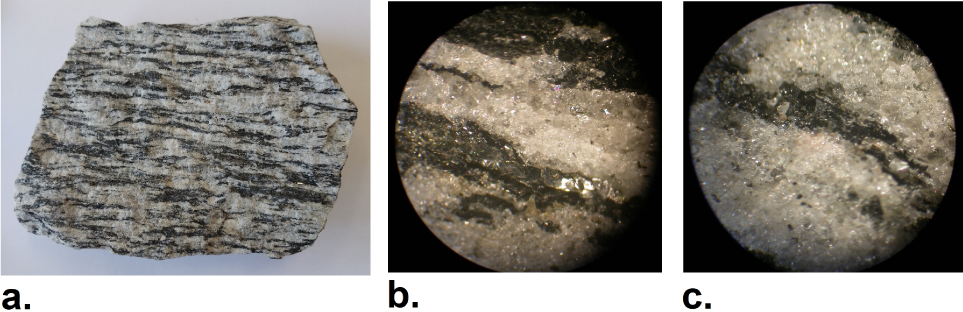
a. Sample of gray gneiss rock. b,c. photomicrographs of regions of the rock sample in a. The dark (black) zones exhibit a uniform tapestry, while the light zones display a crystalline texture (Photograph courtesy of Rabih Sultan and Tamar Kanimian. Copyright 2024).

The geological scenery is a typical landscape where one can find analogies to the diverse features of Liesegang patterns. Although the dynamics may vary from those of the Liesegang scenario, the final pattern often embodies several of its complex morphological characteristics. Given this established reality, the alternation between the compact precipitation bands and the scattered white particles observed in this study, is also expected to find its parallel in rocks.^43^ A relevant example of such an analogy lies in gneiss rocks. Gneiss is a high-grade metamorphic rock presenting an intricate banded pattern, which is a result of an alternation between different minerals aligned as parallel layers.^44,45^ The mechanism of pattern formation in gneiss rocks differs from a typical Liesegang pattern buildup. The banding in gneiss is formed due to a metamorphic transformation of igneous or sedimentary rocks under high-temperature and high-pressure conditions. This gives rise to the recrystallization of the original rock, where the minerals become unstable and rearrange to form new minerals that achieve higher stability under this intense temperature and pressure. This results in the segregation of the minerals into bands, forming alternating layers of different minerals in a direction parallel to that of the applied stress. A sample of gray gneiss is depicted in Figure 6.a.

The dark (almost black) sections are composed mostly of mafic mica minerals, such as biotite, muscovite, or garnet (containing more magnesium and iron), while the light sections are composed of felsic minerals like quartz and feldspar (containing more aluminum, sodium, and potassium). Although gneiss and granite have similar properties (resistance, hardness, durability) and essentially the same composition, they have different textures and finishes; most notably, granite lacks the layered arrangement. The distribution of quartz in light zones consists of an aggregation of crystals (essentially like the Cd(OH)_2_ particles in our system), while the mica spread in the dark zones has a more compact texture rendering. See frames b and c of Figure 6.

## 5 Conclusions

The study described above enabled a rigorous comparison between an experimental Liesegang system involving three precipitates and a theoretical model based on the competitive particle growth (CPG) model. Like most models on Liesegang patterning, the present model considers the generic aspect of the inherent dynamics and does not address the identity of the ions or interaction with the gel. The results may be summarized as follows:

(1) Stability analysis showed that an anticorrelated pattern with an alternation between (*A* + *B*) precipitate bands and zones of single *C* precipitate is always dominant.

(2) Liesegang experiments confirmed an alternation between Co(OH)_2_/Ni(OH)_2_ mixed bands and zones of single Cd(OH)_2_, formed as an aggregate of granular particles.

(3) The obtained Liesegang patterns were compared with banded rock samples, notably gneiss, which could present a plausible analogy.

## 6 Appendix

Stability eigenvalues using model parameters are given in Figure 2.
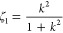